# Psychological inflexibility and resilience in anxiety: insights from machine-learning and robust mediation-based models

**DOI:** 10.3389/fpsyt.2026.1769001

**Published:** 2026-05-18

**Authors:** Ana Ferreira Mostardinha, Daniela Sousa, Hugo Senra, Marco Simões, Ana Pina Rodrigues, Paula Vagos, Miguel Castelo-Branco

**Affiliations:** 1 Coimbra Institute for Biomedical Imaging and Translational Research (CIBIT), Institute for Nuclear Sciences Applied to Health (ICNAS), University of Coimbra, Coimbra, Portugal; 2 Faculty of Medicine (FMUC), University of Coimbra, Coimbra, Portugal; 3 Instituto Superior Miguel Torga (ISMT), Coimbra, Portugal; 4 Institute of Electronics and Informatics Engineering of Aveiro (IEETA), University of Aveiro, Aveiro, Portugal; 5 School of Health and Social Care, University of Essex, Colchester, United Kingdom; 6 Centre for Informatics and Systems (CISUC), Department of Informatics Engineering (DEI), University of Coimbra, Coimbra, Portugal; 7 Center for Research in Neuropsychology and Cognitive-Behavioral Intervention (CINEICC), Faculty of Psychology and Educational Sciences, University of Coimbra, Coimbra, Portugal; 8 William James Research Center, Department of Education and Psychology, University of Aveiro, Aveiro, Portugal

**Keywords:** anxiety, generalized anxiety disorder, psychological inflexibility, resilience, stress

## Abstract

**Introduction:**

Psychological inflexibility (PI) has been associated with anxiety symptoms, while resilience serves as a protective factor; however, their roles and interrelationship remain poorly understood. We investigated the role of PI on anxiety-related symptoms while assessing the mediating role of resilience and testing the moderating effect of sex and psychiatric history.

**Methods:**

From April to July 2021, an online protocol employing self-reported measures assessed PI (Acceptance and Action Questionnaire), resilience dimensions (Resilience Scale for Adults), and anxiety-related symptoms (Generalized Anxiety Disorder (GAD) Scale; Depression, Anxiety, and Stress Scales). A model generation approach, using machine-learning and robust mediation-based models, was applied to investigate the relationships between these constructs.

**Results:**

In a sample of 313 adults (72.20% females; 39.29 ± 11.81 years), Random Forest analysis indicated PI and the resilience dimensions perception of self (R-PS) and planned future (R-PF) as the strongest predictors of anxiety-related symptoms. PI showed a positive direct association with GAD, anxiety, and stress (respectively β = 0.28, β = 0.07, β = 0.20, *p* ≤ 0.001). Significant indirect associations emerged: PI–Stress regarding R-PS (β = 0.08, *p* = 0.004), PI–Anxiety regarding R-PF (β = 0.03; *p* = 0.03), PI–GAD (β = 0.08, *p* = 0.001) and PI–Stress (β = 0.11, *p* < 0.001) regarding R-PS and R-PF together.

**Discussion:**

These findings highlight the importance of PI and resilience as interconnected processes underlying mental health outcomes. Additionally, they suggest that psychological intervention programs targeting PI, along with resilience, could foster healthier strategies for coping with anxiety-related symptoms.

## 1 Introduction

Anxiety is a component of adaptive and expectable responses to stressful situations but, when unregulated, it is also a core feature in several psychiatric disorders (1), being the most prevalent problem in mental health worldwide (2). Anxiety is frequently observed in different settings of healthcare (3), whether as a formal diagnosis of anxiety or as a “subthreshold” condition (4). Anxiety can manifest in cognitive (e.g., attention deficits and/or excessive worrying), affective (e.g., restlessness and/or irritability), physiological (e.g., muscle tension and/or sleep difficulties), and behavioral (e.g., avoidance and/or procrastination) domains (5). These symptoms encompass a preparatory mood state that is activated when future events are anticipated as unpredictable, uncontrollable, and potentially threatening (5).

Anxiety aligns with the stress response and emerges when one perceives available coping strategies to deal with a potential threat as insufficient. Stress is defined as a psychological and physiological response to challenging situations (stressors) that may arise from daily living (e.g., family conflicts or work deadlines) or from extreme negative events (e.g., the loss of a loved one or a pandemic, such as COVID-19) (6). Stress and anxiety are closely connected (7, 8), often overlapping in their manifestations and impact, for example, in reducing concentration and increasing irritability, sleep problems, or avoidance (9). However, they are distinct in the sense that stress is triggered by an external stimulus, whereas anxiety can occur independently of such an external factor (10).

Stress has been particularly linked to Generalized Anxiety Disorder (GAD) (11). GAD is one of the most common anxiety disorders, with lifetime prevalence estimates reaching up to 8% in some countries (12). Characterized by a persistent state of anxiety and expectant concern about a widespread range of topics (e.g., health, family, work), and by the presence of other symptoms (e.g., difficulty concentrating, irritability, muscle tension, and sleep difficulties) (1), GAD has a significant impact on quality of life (13). Additionally, this disorder is often comorbid with medical conditions, such as migraine (14), and with other psychiatric diagnoses, especially mood and other anxiety disorders (12). Due to the overlapping symptoms with other diagnoses, including anxiety and mood comorbidities, GAD is considered by some authors as a shared pathway or a subjacent entity to anxiety-related problems (15).

Over time, research has explored what protects individuals against anxiety-related symptoms from early in life (16–20). Resilience is widely recognized as a protective factor for anxiety (21) and has been increasingly targeted in intervention programs (22). Resilience is the ability to function adaptatively, by activating protective resources, in the face of significant adversity and stress (23). It refers to a multidimensional concept, including the perception of the self, which concerns individual psychological competencies (e.g., internal locus of control and optimism for the future), the perception of family harmony (e.g., cooperation and conflict), and the perception of one’s external systems as sources of support (e.g., relatives and friends’ availability) (23). Leys et al. (24) found evidence suggesting that resilience protects against significant stressful events, reducing anxiety-related symptoms at the time of exposure and promoting recovery three months later. In other words, resilience appears to aid in managing both immediate difficult experiences and contributes to sustained mental well-being.

In turn, psychological inflexibility (PI) has been proposed as a common component in anxiety disorders (25) and a core transdiagnostic process implicated in several mental health problems (26), being targeted by Acceptance and Commitment Therapy (ACT). ACT is a third-generation cognitive-behavioral therapy that is grounded in Relational Frame Theory, a behavioral theory of human language and cognition. RFT proposes that individuals learn to relate events/stimuli through arbitrarily applicable relational responding, which in turn influences emotions and behavior. This can lead to suffering and psychopathology, if internal events are seen as absolute truths and rigidly avoided (27). So, according to ACT/RFT, PI consists of a rigid pattern of control and avoidance of negative and uncomfortable private experiences (such as thoughts, emotions and bodily sensations), leading to a reduced capacity to act in accordance with core personal values, thereby resulting in distress and even psychopathology (27). Interventions based on third-wave therapies, such as ACT, focus on helping individuals relate to their thoughts and emotions (28) through psychological flexibility (PF), thus countering PI (27). As stated by this approach, PI is maintained by six interrelated processes, which could be modified by enhancing the six interrelated processes that support PF. The processes underlying PI (with the corresponding PF processes in brackets) are as follows: cognitive fusion (cognitive defusion), experiential avoidance (acceptance), attachment to the conceptualized self (self-as-context), conceptualized past/feared future (contact with the present moment), lack of values clarity (values clarity), unworkable and not value-guided actions (committed action) (for more detailed description (27, 29). Recent studies have consistently demonstrated that PI is strongly linked to anxiety-related disorders (30, 31). Specifically, PI has shown positive associations with stress, GAD symptoms, and excessive worry (32). A study from Roemer et al. (33) involving both clinical and non-clinical samples of individuals with GAD symptoms, indicates that the tendency to avoid internal experiences is closely associated with worry and the severity of GAD symptomatology, and predicts GAD severity even after controlling for the effects of worry itself.

It thus seems that PI and resilience are inversely related both conceptually and in terms of their impact on anxiety-related symptoms. Interestingly, despite this clear conceptual opposition, there is a lack of studies that examine the specific role and relationship between PI and resilience in anxiety. Previous research has adopted different approaches, with several studies examining PI and resilience as potential mediators in the relationship between other constructs – such as between attachment and well-being [e.g., (34, 35)]. Other studies have investigated the mediating role of the tendency to avoid/control unwanted private events in the relationship between anxiety and quality of life (36), and the mediating effect of resilience in the relation between anxiety and mindfulness and self-compassion (37).

It remains, however, unclear to what extent PI and resilience are related, the nature of this relationship, and its implications on the symptomatology of anxiety. Given the growing evidence that both PI and resilience are positively and negatively associated with anxiety-related symptoms, respectively (21, 32), in line with the theoretical connection between PI and resilience, we propose to investigate the role of both on a explanatory model of psychological vulnerability to anxiety. Understanding individual psychological processes, along with how these elements interact, is key to developing more effective interventions. Furthermore, understanding potential sex differences in these processes is important, given the increased vulnerability observed in women. Specifically, being a female has been associated with an increased risk of developing anxiety-related symptoms and their severity (38), alongside with generally lower resilience levels (39), and higher levels of PI (40).

In sum, we aimed to investigate 1) the predictive importance and relative role of PI and resilience on anxiety-related symptoms; 2) the relationship between PI and anxiety-related symptoms, with resilience serving as a mediating factor, adjusting for clinical and demographical relevant variables (namely sex, age, and history of psychiatric diagnosis); and finally, 3) the potential moderating effects of sex and history of psychiatric diagnosis on the previously mentioned mediation models.

## 2 Materials and methods

The study was approved by the Ethics Committee of the Faculty of Medicine of the University of Coimbra (ethical approval number: CE-046/2020). Informed consent was obtained from the participants, after an explanation of the nature of the study. This study was designed, conducted and reported to promote transparency and reproducibility.

### 2.1 Study design and participants

The present work is a cross-sectional, observational, online survey study. Data collection took place between April and July 2021 through an online self-report protocol, which was part of a larger survey conducted on the Neurohab platform. Neurohab is a software platform developed by our research team, to support scientific research and specially designed for the intervention and rehabilitation of patients that can also be used by caregivers and therapists (41). Participants were recruited using a convenience sampling technique, while using a wide dissemination strategy. The self-report protocol link was disseminated through the institutional volunteer database, social media platforms, and webinar presentations organized by the research team. Eligible participants were adults aged 18 years or older, resident in Portugal. Participants were excluded if they were under 18 years old or resided outside Portugal.

### 2.2 Measures

The sociodemographic questionnaire included demographic, professional, and psychiatric variables. The self-report protocol included questionnaires that assessed psychological inflexibility, resilience, and GAD, anxiety, and stress symptoms.

Psychological inflexibility was assessed using the Acceptance and Action Questionnaire 2 (42, 43) (AAQ-2). The scale consists of 7 statements that reflect the individual’s inflexibility in dealing with difficult events, thoughts, and feelings, answered on a 7-point Likert scale (from 1 = never true to 7 = always true). The total score ranges from 7 to 49, with higher scores indicate higher levels of psychological inflexibility. In this work, the scale presented a high level of internal consistency (*α* = 0.94), in line with the results of Pinto-Gouveia et al. (43) (*α* = 0.89). Moreover, these authors found construct validity, concerning measures that assess psychopathological symptoms (such as Depression, Anxiety, and Stress Scales) and adaptative and dysfunctional emotional regulation processes.

Resilience was measured by the Resilience Scale for Adults (44, 45) (RSA). The scale consists of 33 items related to resources that promote resilience across six dimensions: perception of self, planned future, social competence, structured style, family cohesion, and social resources. Items are answered on a 7-point Likert scale (from 1 to 7, with item-specific response meanings). The total score ranges from 33–231, higher scores indicating higher levels of resilience. Subscales include different numbers of items, therefore score ranges differ across dimensions. In our work, the subscales demonstrated at least acceptable internal consistency (*α* ranging from 0.78 to 0.85, except the Structured Style subscale, *α* = 0.48). Pereira et al. (45) reported similar internal consistency coefficients (*α* ranging from 0.72 to 0.84, except the Structured Style subscale, *α* = 0.38). Furthermore, their findings indicated that participants with psychiatric illness had significantly lower values in resilience and all its dimensions.

GAD symptoms experienced in the previous two weeks were assessed using the Generalized Anxiety Disorder Scale (46, 47) (GAD-7). The 7 items correspond to the DSM-IV diagnostic criteria and are answered on a 4-point Likert scale (from 0 = never to 3 = nearly every day), assessing the severity of symptoms related to GAD. Score range between 0 to 21, with higher values indicating greater symptom severity. The scale presented excellent reliability in our work (*α* = 0.94), consistent with the findings of Sousa et al. (47) (*α* = 0.88). Moreover, their study provided evidence for construct validity, as shown by significant correlations with other measures of anxiety, such as the Hospital Anxiety and Depression Scale.

Anxiety and stress symptoms experienced in the past week were measured by the Depression, Anxiety, and Stress Scales (48, 49) (DASS-21). The scale consists of 21 items that refer to negative emotional symptoms answered on a 4-point Likert scale (from 0 = did not apply to me at all to 3 = applied to me most of the time), assessing three dimensions of psychopathology symptoms: depression, anxiety, and stress. Total score ranges from 0–63 and subscales from 0–21. Higher scores indicate greater overall psychological distress and greater severity of the respective symptom domains (depression, anxiety, and stress). We found a good internal consistency in the two subscales that were considered in the current work (*α* = 0.86 on the anxiety scale and 0.92 on the stress scale), consistent with the results reported by Pais-Ribeiro et al. (49) (*α* = 0.74 on the anxiety scale and 0.81 on the stress scale).

### 2.3 Statistical analysis

Data was analyzed with the IBM Statistical Package for the Social Sciences (SPSS), Version 27, specifically for primary descriptive analysis, and the R software (version 4.3.1; RStudio 2023.12.0) for the remaining analyses.

All data were verified for missing cases, incorrect values, symmetry of dependent variables distribution (normality), and extreme values (regarded as outliers when *Z*-score ≥ 3). Pearson correlation coefficients were calculated to examine the bivariate associations between the study variables. Statistical significance was set at *p* < 0.05.

The following analysis approaches were undertaken: 1) Machine learning (ML)- based models to investigate the predictive importance of our independent variables (age, sex, history of psychiatric diagnosis, PI, and dimensions of resilience) on the dependent variables (symptoms of GAD, anxiety, and stress); 2) Robust Mediation Models to test associations between the independent variable PI and the dependent variables, through the mediating effect of the dimensions of resilience that were suggested by the ML models to have greater predictive importance on the dependent variables; 3) Moderated Mediation Models to study a possible moderation effect of sex and the history of psychiatric diagnosis on the previously mentioned mediation models using one single mediator, based on robust mediation models results. The theoretical mediation and moderated mediation models are presented in **Supplementary Materials** (**Supplementary Figures S1**, **S2**).

Regarding ML models, given the study sample size, we decided to use regression ML algorithms that are described in the literature as more appropriate for smaller samples (50, 51), specifically 1) Random Forest, 2) Elastic Net, 3) Gradient Boosting, and 4) Extreme Gradient Boosting. Before starting each ML Model, a Boruta algorithm was run with all study variables, for feature selection purpose. Only non-rejected predictors according to Boruta results have been included in the ML models to increase their performance. ML models have been run with a total of nine normalized predictors. Data partition included ten random dataset splits (70% for training and 30% for test), to reduce chances of bias coming from a single data partition. Hyperparameter tuning was performed using k = 10-fold cross validation and using the lowest RMSE as the selection criteria. Hyperparameters tunned included alfa and lambda for Elastic Net; number of trees and mtry for Random Forest; number of trees, learning rate, maximum tree depth, and minimum sum of instance weight for Gradient Boosting; eta and maximum depth for Extreme Gradient Boosting. Default values have been used for the remaining hyperparameters. ML models with the best performance (lowest RMSE) were taken to inform which features should be included in the mediation models. ML models were performed with the R packages caret, gmb, glmnet, and xgboost.

Robust mediation models as described by Alfons et al. (52) were adopted considering the existence of extreme values and skewed distributions of the dependent variables, and the fact that the statistical assumptions for linear regression had not been fully met (errors not normally distributed). This method combines the robust MM-estimator of regression as described by Yohai (53) with the fast and robust bootstrap techniques. Robust mediation models were run using the robmed R package. For each dependent variable, we tested a robust mediation model with the inclusion of sociodemographic co-variables (age and sex, for example) suggested as important by the ML algorithms. All robust mediation models have been run with 5000 bootstrap replicates. Finally, the moderated mediation models include two binary variables as moderators, “sex” and “history of psychiatric diagnosis”. As these variables were heavily unbalanced for the number of cases in each category, Propensity Score Matching procedures, using the Optimal Full Matching method, were undertaken to reduce the potential bias effect of these variables in the mediation analysis. Moderated mediation models were performed with the mediation R package, using non-parametric bootstrap methods, and a quasi-Bayesian approximation (with 1000 Monte-Carlo draws).

Sample size calculations were undertaken considering the statistical methods planned for this research. For the ML models, the literature based on observed and simulated analysis suggested that the performance error (e.g. RSME) tends to stabilize at a minimum sample size of 200 individuals for the training set (54, 55), which makes a minimum sample size of 285 individuals (70% for the training set) required for ML models. Sample size and power calculations for causal mediation models were undertaken for a power (1-*β*) ranging from 0.8 to 0.9, an estimated effect size (standardized regression coefficient) of 0.2, and two covariates, using the Causal Mediation Power Analysis shiny app, which employs a power analysis method based on Monte-Carlo simulations (Qin, 2024). Simulation results (1000 Monte-Carlo draws) suggested a required sample size ranging from 276 (1-*β* = 0.8) and 333 (1-*β* = 0.9).

## 3 Results

### 3.1 Participants

We reached a sample of 313 adults (72.20% females), with a mean age of 39.29 years (standard deviation, SD = 11.81 years, ages ranging from 18 to 75 years). A total of 67 (21.41%) participants reported having a past/present psychiatric diagnosis (depression [N = 33], anxiety [N = 23], obsessive-compulsive disorder [N = 5], bipolar disorder [N = 2], and attention deficit hyperactivity disorder, eating disorder, post-traumatic stress disorder, and psychotic and depressive episode [N = 1 for each]) (see **Table 1**).

**Table 1:** Participants’ sociodemographic data and history of psychiatric diagnosis.

Characteristic	Participants (*N* = 313)
Sex, *N* (%)	
Female	226 (72.20)
Male	87 (27.80)
Age in years, Mean, *M* (*SD)*	39.29 (11.81)
Civil status, *N* (%)	
Single/Separated	66 (21.09)
In a relationship	61 (19.49)
Marital/non-marital partnership	157 (50.16)
Divorced	29 (9.27)
Education, *N* (%)	
≤ 9° grade	10 (3.19)
12° grade	54 (17.25)
Degree	127 (40.58)
Master’s degree	94 (30.03)
Doctorate	23 (7.35)
Bachelor’s degree	1 (0.32)
Other (e.g., technical training)	4 (1.28)
Professional condition, *N* (%)	
Student	43 (13.74)
Employed	228 (72.84)
Unemployed	33 (10.54)
Retired	9 (2.88)
If has had a psychiatric diagnosis, *N* (%)	
No	246 (78.59)
Yes	67 (21.41)
If yes, received psychological/psychiatric/psychotherapeutic treatment, *N* (%)	
No	9 (13.43)
Yes	58 (86.57)
If yes, received pharmacological treatment, *N* (%)	
No	7 (10.45)
Yes	60 (89.55)

M, Mean; SD, Standard Deviation; N, Number of participants.

**Table 2** presents the descriptive statistics (mean and standard deviation) for the study questionnaires, reported for the overall sample and separately by sex and history of psychiatric diagnosis. See **Supplementary Figure S3** and **Supplementary Table S1** in the **Supplementary Materials** for the Pearson correlation coefficient values illustrating the bivariate associations between the study variables.

**Table 2:** Participant’s characterization by questionnaires, M (SD).

Variable (questionnaire)	All (*N* = 313)	Males (*N* = 87)	Females (*N* = 226)	Without a history of psychiatric diagnosis (*N* = 246)	With a history of psychiatric diagnosis (*N* = 67)
Psychological inflexibility (AAQ-2)	20.75 (10.10)	18.72 (9.32)	21.53 (10.30)	19.67 (9.67)	24.72 (10.73)
Resilience (RSA)	166.56 (27.97)	164.56 (29.42)	167.32 (27.42)	168.99 (26.53)	157.63 (31.35)
Perception of self	28.66 (6.92)	29.69 (6.83)	28.26 (6.93)	29.19 (6.77)	26.70 (7.17)
Planned future	18.68 (5.57)	19.25 (5.51)	18.46 (5.58)	18.91 (5.35)	17.81 (6.27)
Social competence	29.51 (6.71)	29.44 (6.59)	29.54 (6.77)	29.87 (6.63)	28.16 (6.89)
Structured style	18.94 (4.07)	18.00 (4.08)	19.31 (4.02)	19.16 (4.07)	18.15 (4.03)
Family cohesion	31.75 (6.64)	30.33 (7.21)	32.30 (6.34)	32.34 (6.06)	29.60 (8.14)
Social resources	39.02 (7.51)	37.85 (7.47)	39.47 (7.50)	39.51 (6.99)	37.21 (9.03)
Generalized Anxiety Disorder symptoms (GAD-7)	6.38 (5.38)	4.71 (4.31)	7.02 (5.62)	5.86 (5.34)	8.28 (5.14)
Anxiety symptoms (DASS-21-A)	2.73 (3.59)	1.98 (2.81)	3.01 (3.81)	2.25 (3.18)	4.48 (4.39)
Stress symptoms (DASS-21-S)	5.95 (4.86)	4.49 (3.98)	6.50 (5.06)	5.30 (4.70)	8.31 (4.75)

M, Mean; SD, Standard Deviation; N, Number of participants; AAQ-2, Acceptance and Action Questionnaire 2; RSA, Resilience Scale for Adults; GAD-7, Generalized Anxiety Disorder Scale; DASS-21-A, Depression, Anxiety, and Stress Scales – Anxiety scale; DASS-21-S, Depression, Anxiety, and Stress Scales – Stress scale.

### 3.2 Machine learning-based models

Boruta algorithm results suggested the following independent variables to be rejected as predictors of the dependent variables: family cohesion resilience dimension for GAD and stress symptoms; structured style resilience dimension for anxiety symptoms; and age for stress symptoms. The remaining independent variables were therefore included in the ML-based models for the three dependent variables. The results of ML models are presented in **Table 3**. Full results of Boruta algorithm for all dependent variables (**Supplementary Tables S2–S4**; **Supplementary Figures S4–S6**), including the resulting variables selection (**Supplementary Table S5**), are provided in the **Supplementary Materials**.

**Table 3:** Machine learning-based models results for the dependent variables.

*Predictors*	Generalized anxiety disorder symptoms (GAD-7)				Anxiety symptoms (DASS-21-A)				Stress symptoms (DASS-21-S)			
	Elastic net	Random forest*	Gradient boosting	Extreme gradient boosting	Elastic net	Random forest*	Gradient boosting	Extreme gradient boosting	Elastic net	Random forest*	Gradient boosting	Extreme gradient boosting
	*Estimates*	*Variable importance*	*Relative influence*	*Gain*	*Estimates*	*Variable importance*	*Relative influence*	*Gain*	*Estimates*	*Variable importance*	*Relative influence*	*Gain*
Age	0.00	14.56	1.92	0.11	0.00	20.23	3.28	0.11				
Sex	0.63	7.80	2.76	0.02	0.00	16.00	0.05	0.02	1.55	4.40	4.28	0.02
History of psychiatric diagnosis	0.03	5.11	0.91	0.02	1.60	75.22	11.79	0.02	0.79	6.06	1.45	0.03
Psychological Inflexibility (AAQ-2)	0.22	100.00	56.93	0.47	0.13	100.00	51.29	0.47	0.16	100.00	46.72	0.45
Perception of self (RSA)	-0.09	48.61	13.92	0.08	-0.03	67.18	15.45	0.08	-0.13	31.08	18.95	0.17
Planned future (RSA)	-0.03	48.18	12.10	0.08	-0.06	57.68	7.73	0.08	-0.13	32.19	14.94	0.10
Social competence (RSA)	0.00	14.08	5.55	0.10	0.00	0.00	2.36	0.10	0.01	3.33	3.77	0.07
Structured style (RSA)	0.00	13.20	1.87	0.09					0.05	0.00	2.25	0.08
Family cohesion (RSA)					0.00	33.44	2.15	0.09				
Social resources (RSA)	-0.01	0.00	4.03	0.05	-0.01	45.38	5.92	0.05	-0.08	10.66	7.64	0.07
RMSE (95% CI)	17.15 (15.50– 18.80)	17.55 (16.00 – 19.10)	18.29 (16.84 – 19.72)	26.50 (20.95 – 32.06)	8.74 (8.28 – 9.21)	8.65 (8.05 – 9.26)	9.26 (8.80 – 9.72)	13.02 (10.55 – 15.51)	13.27 (12.06 – 14.49)	13.41 (11.99 – 14.83)	14.14 (12.75 – 15.53)	19.03 (16.85 – 21.22)
R^2^ (95% CI)	0.45 (0.40 – 0.49)	0.43 (0.38 – 0.47)	0.40 (0.36 – 0.44)	0.25 (0.14 – 0.36)	0.31 (0.26 – 0.36)	0.32 (0.26 – 0.39)	0.27 (0.23 – 0.31)	0.21 (0.08 – 0.34)	0.31 (0.26 – 0.36)	0.32 (0.26 – 0.39)	0.27 (0.23 – 0.31)	0.21 (0.08 – 0.34)

GAD-7, Generalized Anxiety Disorder Scale; DASS-21-A, Depression, Anxiety, and Stress Scales – Anxiety scale; DASS-21-S, Depression, Anxiety, and Stress Scales – Stress scale; AAQ-2, Acceptance and Action Questionnaire 2; RSA, Resilience Scale for Adults. * Mean decrease accuracy used in Random Forest models.

The Elastic Net and Random Forest models showed the best performance, with the lowest RMSE values for all dependent variables. However, the Random Forest performed better than Elastic net for differentiating the importance of each psychological variable, including each resilience dimension, and was therefore chosen for feature selection purposes.

#### 3.2.1 Generalized anxiety disorder symptoms

In Random Forest results, PI and resilience dimensions perception of self and planned future were the psychological predictors of generalized anxiety disorder symptoms with the highest importance. Within the sociodemographic covariates, age showed the greatest importance (see **Table 3**).

#### 3.2.2 Anxiety symptoms

In Random Forest results, PI and resilience dimensions perception of self and planned future were the psychological predictors of anxiety symptoms with the highest importance. Within the sociodemographic covariates, history of psychiatric diagnosis showed the greatest importance (see **Table 3**).

#### 3.2.3 Stress symptoms

In Random Forest results, PI and resilience dimensions perception of self and planned future were the psychological predictors of stress symptoms with the highest importance. Within the sociodemographic covariates, sex and history of psychiatric diagnosis showed the greatest (and similar) importance (see **Table 3**).

### 3.3 Robust mediation models

**Figure 1:**
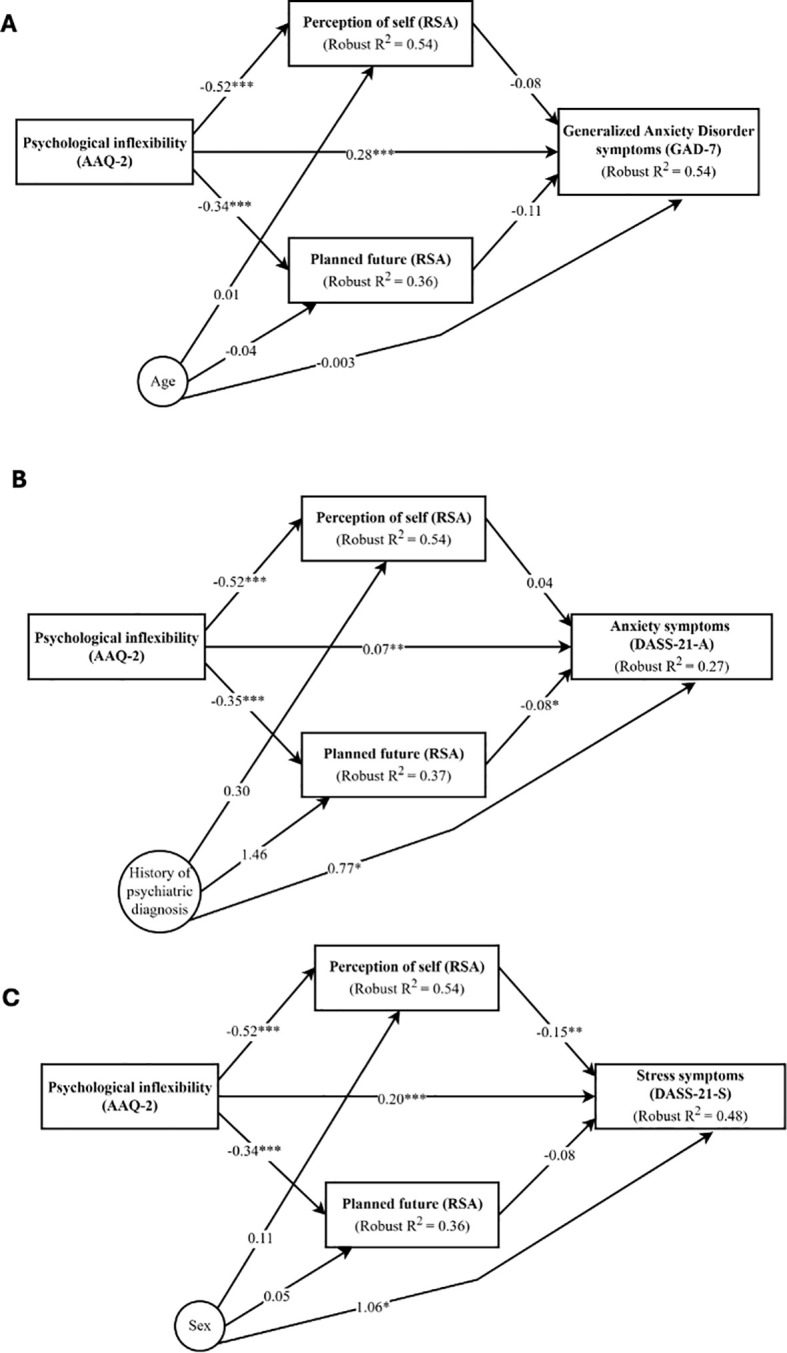
Schematic representation of the mediation models. **(A)** Mediation model for Generalized Anxiety Disorder symptoms (GAD-7). **(B)** Mediation model for anxiety symptoms (DASS-21-A). **(C)** Mediation model for stress symptoms (DASS-21-S). Values refer to coefficients for each regression path. Full results for total and individual indirect effects are reported in **Supplementary Tables S6–S8** in the **Supplementary Materials**. ***p  < 0.001, **p < 0.01, *p  < 0.05 for reporting statistical significance for each regression path in the mediation model.

Parallel mediation models were undertaken according to results from ML-based models, which suggested two resilience dimensions, perception of self and planned future, to have the highest predictive importance for all outcome variables, adjusting for demographic and/or clinical covariates (age, sex, history of psychiatric diagnosis). The robust mediation model for stress symptoms was run only with sex as sociodemographic covariate, because the algorithm did not converge with the other alternatives: sex and history of psychiatric diagnosis, or history of psychiatric diagnosis as the only covariate. The mediation models are presented in **Figure 1**. The full statistical results for all mediation models are presented in **Supplementary Materials** (**Supplementary Tables S6–S8**).

#### 3.3.1 Generalized anxiety disorder symptoms

The mediation model showed a significant direct effect between PI and symptoms of GAD (Boot β = 0.28; 95% Confidence Intervals, CI: 0.04-6.98, *p* < 0.001). A significant total indirect effect was found between PI and GAD symptoms, via the sum of the two indirect effects (through the two mediators: perception of self and planned future resilience dimensions) (Boot β = 0.08; 95% CI: 0.03-0.13, *p* = 0.001) (see **Supplementary Table S6** in **Supplementary Materials**). The model, therefore, suggests that PI is associated with greater levels of GAD symptoms, with higher scores on the two resilience dimensions together reducing the positive association between PI and GAD symptoms. However, none of the resilience dimensions (perception of self and planned future) individually showed a significant mediating effect, which suggests that the association between PI and GAD symptoms is only mediated by the effect of both mediators together but not by any of them individually. The covariate age was not significantly associated with the mediators nor the dependent variable (see **Figure 1A**).

#### 3.3.2 Anxiety symptoms

The mediation model showed a significant direct effect between PI and symptoms of anxiety (Boot β = 0.07; 95% CI: 0.02-3.18, *p* = 0.001), and a significant individual mediation effect of the variable planned future resilience dimension on the indirect association between PI and symptoms of anxiety (Boot β = 0.03; 95% CI: 0.004-0.06; *p* = 0.03). Specifically, PI is negatively associated with the dimension of the resilience planned future (Boot β = - 0.35; *p* < 0.001) which in turn is negatively associated with symptoms of anxiety (Boot β = - 0.08; *p* = 0.03). The model, therefore, suggests that PI is associated with greater symptoms of anxiety, with higher scores on planned future reducing the positive association between PI and anxiety symptoms. No significant total indirect effect was found suggesting a mediation effect for the sum of the two resilience dimensions (see **Supplementary Table S7** in **Supplementary Materials**). The covariate of history of psychiatric diagnosis was significantly associated with the dependent variable (anxiety symptoms) (see **Figure 1B**).

#### 3.3.3 Stress symptoms

The mediation model showed a significant direct effect between PI and symptoms of stress (Boot β = 0.20; 95% CI: 0.04-4.87, *p* < 0.001), plus a significant total indirect effect between PI and symptoms of stress, via the sum of the two indirect effects (through the two mediators: perception of self and planned future resilience dimension) (Boot β = 0.11; 95% CI: 0.06-0.16, *p* < 0.001) (see **Supplementary Table S8** in **Supplementary Materials**). The perception of self was found to individually mediate the association between PI and symptoms of stress (Boot β = 0.08; 95% CI: 0.03-0.14, *p* = 0.004). The mediation model suggests that PI is negatively associated with perception of self resilience dimension (Boot β = - 0.52; *p* < 0.0001) which in turn is negatively associated with symptoms of stress (Boot β = - 0.15; *p* = 0.006). The model also suggests that the two mediators together have the same negative effect on the positive association between PI and symptoms of stress (see **Figure 1C**).

### 3.4 Moderated mediation models

Moderated mediation models were undertaken to test the hypothesis that history of psychiatric diagnosis and sex (separately) are moderators of the mediation effect that perception of self can have on the association between PI and mental health outcomes (symptoms of GAD, anxiety, and stress). Only the variable perception of self resilience dimension was included as theoretical mediator, as it was the variable identified with the greatest importance in the ML models, and to avoid moderated models with high complexity. Full results are presented in **Supplementary Materials** (**Supplementary Table S9**).

All moderated mediation models both with history of psychiatric diagnosis and sex as moderators showed non-significant (*p* > 0.05) average causal mediation effect (ACME) estimates. Tests for ACME differences between moderator levels showed *p*-values greater than 0.05, which suggests that the mediators are not significantly influenced by the moderators, i.e., there is no evidence of significant moderated mediation models for our dependent variables (see **Supplementary Table S9** in **Supplementary Materials**).

## 4 Discussion

In this study, after investigating the predictive power of psychological inflexibility (PI) and resilience dimensions on anxiety-related symptoms, we directly asked how they interact and contribute to explaining the experience of those symptoms. We also addressed the question of whether the association of these processes with anxiety-related symptoms is manifested differently in men/women and individuals with/without a history of psychiatric diagnosis. The current study proposed a hypothetical vulnerability framework based on multivariate association patterns concerning anxiety-related symptoms. These findings shed light on the mechanisms underlying anxiety and ultimately inform psychological interventions designed to prevent or treat these symptoms. Although advanced statistical procedures were adopted, the cross-sectional and self-report nature of the study means that the results should be interpreted as reflecting relationship associations rather than causal effects.

PI and resilience (specifically the perception of self and planned future dimensions) were relevant statistical predictors of GAD, anxiety and stress symptoms, with PI showing a risk association and resilience presenting a protective relation. However, when adjusting for other variables, PI appears as the most statistically important predictor within the model of all anxiety-related symptoms (symptoms of GAD, anxiety and stress). Several studies have identified a link between PI and anxiety symptoms. For instance, Krafft et al. (31) found a strong correlation between reduced worry and changes in PI following an ACT-based intervention that targeted PI in individuals with GAD, although a causal relationship was not established. Additionally, other studies have demonstrated the predicted power of PI on anxiety-related symptoms in clinical populations, such as veterans with posttraumatic stress disorder (56). Our study is consistent with these previous findings in specific groups, while also providing additional significant insights concerning the strength of relationships considering different predictors in a community sample. Specifically, after controlling for relevant variables, PI showed stronger statistical associations with anxiety symptoms than resilience dimensions.

In line with these findings, we ran robust mediation models which showed that the link between PI and anxiety-related symptoms was found to be statistically mediated by resilience. Namely, perception of self seems to mediate the relationship between PI and symptoms of stress, while planned future appears to mediate the association between PI and symptoms of anxiety. Together, perception of self and planned future, statistically mediated the link of PI with GAD and stress symptoms. In general, PI may have a risk association with levels of GAD, anxiety and stress symptoms, which could be hypothetically suppressed by self- and future-perception. Despite the clear theoretical relationship between PI and resilience, there is a lack of literature exploring this connection. Some research focuses on psychological flexibility (PF) as a resilience resource in itself [e.g., (57, 58)]. Conversely, literature has been consistently relating resilience and anxiety [e.g., (21)], including evidence of interventions to foster resilience (22). Our study provides new insights into the interaction between PI and specific domains of resilience and goes one step forward, by advancing the understanding of how individuals’ perceptions of their ability to cope with challenges is associated with the experience of anxiety-related symptoms, clarifying resilience specific aspects that show higher predictive contribution in the development of these symptoms. The perception of self and planned future are conceptually two core RSA subscales related to the individuals’ perception of their own personal skills that help them cope with both present and future situations; the remaining subscales focus on more specific aspects (e.g., behavioral organization) and/or involve interpersonal resources. Inclusively, these two subscales were previously combined into one, which focused on personal strength, and were identified as key resource indicators that can help work against psychological vulnerability (59).

According to ACT, PI is manifested by an individual getting stuck in the literal content of negative thoughts, making efforts to control and avoid private difficult experiences (in the form of thoughts, feelings, or bodily sensations), leading to suffering and limited adaptative behavior (27). Often, these thoughts include self-conceptualizations that involve negative experiences and beliefs about oneself, that contribute to PI (27). These self-conceptualizations could theoretically be equivalent to the perception of self dimension of resilience in the RSA, which focuses on individuals’ confidence in their abilities and judgments, self-efficacy, and the alignment of expectations with reality (45). Indeed, Jeffords et al. (60) identified a significant direct association between PI and college self-efficacy among university students, in a particular context of stigma. Furthermore, some authors have found a significant inverse correlation between self-efficacy and anxiety (61, 62), i.e., individuals who perceive themselves as less capable also seem to feel more anxiety.

In turn, the planned future dimension of resilience in the RSA reflects the individuals’ positive view about the future, beliefs of success, and the ability to plan, set, and be guided by clear and achievable goals in advance (45). According to Hayes et al. (29), PI impacts on one’s actions, leading to a life dominated by a focus on the past and future, i.e., individuals psychologically inflexibly tend to get stuck and try to avoid/control negative thoughts relative to the past/future. Ranjbar et al. (63) suggested that PI processes functioned as mediators of the relation between negative thoughts and feelings relative to past/present and anxiety. In this regard, main anxiety conditions, such as GAD, are characterized by negative future-oriented thinking [see the review of Moustafa et al. (64)].

Finally, we explored the potential statistical moderator role of sex and history of psychiatric diagnosis in the identified indirect and direct relationships. Considering the higher prevalence of PI [e.g., (40, 65)] and lower levels of resilience (39, 66) in these groups, it could be expected that the link of PI with anxiety-related symptoms, statistically mediated by resilience, would be stronger in women and those with a history of mental health problems. Conversely, our results provide an indication that, regardless of sex and psychiatric diagnostic history, individuals present a consistent pattern of associations among psychological processes. Our findings support the hypothesis that PI is a core transdiagnostic feature of mental health problems, regardless of their nature (26, 67). However, the present findings should be interpreted with caution, avoiding conclusions regarding these association patterns, given the study design and the relatively large group sample sizes that are required for multivariate analyses. Further studies are recommended to replicate and extend these analyses.

Overall, taking our results together, we propose that PI plays a relevant predictive role in the presence and development of anxiety-related symptoms, and resilience dimensions could mitigate its associations. So, in other words, we argue that possibly higher levels of anxiety are not only associated with larger psychological inflexibility but also with this inflexibility manifesting itself in lower resilience associated with how we perceive ourselves and the future. This could be particularly impactful for the development of psychological intervention programs. As we have argued, our findings are theoretically consistent with the conceptual foundations of ACT. In fact, ACT conceptualizes suffering/psychopathology as resulting from PI, while well-being/psychological health as resulting from PF (27). Accordingly, ACT-based interventions aim to improve PF by reducing PI, focusing on changing the individual’s relationship with difficult internal experiences (27) rather than analyzing and/or changing their content (which is the target of Cognitive and Behavioral Therapy) (28). This ACT-based approach has demonstrated effectiveness across several psychological difficulties, including anxiety, tended to outperform inactive control conditions, such as treatment as usual, and most intervention control conditions (68). According to Hayes et al. (27, 29), individuals tend to become cognitively fused with two major themes: beliefs about themselves, such as their identity, roles, skills and worth (i.e., the conceptualized self) and negative interpretations and self-evaluations relative to past/future experiences (i.e., conceptualized past/feared future). This is also supported by our machine-learning models, which identified perception of self and planned future resilience dimensions as the most statistically important study variables predicting anxiety-related symptoms, after PI. Moreover, in our study, these variables also appeared to play a statistical mediating role in the relationship between PI and anxiety-related symptoms. In other words, by changing the individual’s relationship with difficult internal experiences, particularly those related to personal (i.e., conceptualized self/perception of self) and future content (i.e., conceptualized future/planned future), psychological openness to negative internal experiences may be promoted (i.e., increasing PF and reducing PI), that may be associated with lower anxiety symptoms. From a practical perspective, ACT-based intervention programs may benefit from incorporating modules specifically designed to target PF/PI (through their processes), applied to self-perception (e.g., self-efficacy, confidence in coping with challenges) and future-oriented thinking (e.g., realistic and adaptive planning).

Despite the potential significance of this study, the findings should be interpreted considering both its methodological strengths and limitations. First, the cross-sectional design of the study gives us the strength and direction of variables’ associations at a specific point, which provides valuable insight into their relationships. However, longitudinal or experimental designs would be necessary to analyze the stability of these associations over time and to draw causal inferences. Specifically, regarding intervention research, it would be particularly relevant to conduct a randomized controlled trial comparing ACT-based programs with and without additional sessions explicitly designed to target self- and future-perception in groups with clinical and subclinical levels of anxiety. Second, the exclusive reliance on self-report measures may have introduced response biases (e.g., social desirability), potentially affecting internal validity, despite the use of validated and well-established instruments to assess the study variables. Moreover, the recruitment strategy and the sample characterization (e.g., only people residing in Portugal) may limit the external validity of the findings, i.e., the generalizability of the findings to other populations or cultural contexts. However, the use of the convenience sampling technique enabled efficient recruitment of a sufficiently large sample to address the study’s objectives of considering the role of multiple variables. Additionally, the sample’s specificity provides meaningful insights into the pattern of associations analyzed within a clearly defined cultural context, supporting future cross-cultural research. A key methodological strength of this study is its theory-driven design, which guided both the selection of variables and the analytical strategy. Additionally, the advanced statistical procedures adopted allowed the examination of both predictive performance and indirect pathways among variables, providing a more robust and comprehensive analytical approach to underlying mechanisms. However, although we followed methodological recommendations for applying machine learning models to small samples [e.g (51)], the present findings should be interpreted with caution. The relatively small sample size when considering the complexity of the analysis may still limit the parameter estimates precision and the detection of small effect sizes. In the same line, although we have taken some procedures to mitigate the loss of balance between the group’s sizes (i.e., men/women and individuals with/without a history of psychiatric diagnosis), we recommend testing the moderated mediation models in more balanced samples. Finally, it is important to acknowledge that data collection occurred between April and July 2021, during the exceptional psychosocial context of the COVID-19 pandemic, which has influenced levels of anxiety (69), and possibly PI and resilience-related processes. Although this is a limitation, and should be considered when interpreting our findings, it also provides insight into the association patterns among these psychological variables during a particularly challenging period. Therefore, future studies are encouraged to replicate these results in non-pandemic and in longitudinal samples to further validate the stability of these associations.

## Data Availability

The raw data supporting the conclusions of this article will be made available by the authors, without undue reservation.
