# Supplementary material for: Psychological inflexibility and resilience in anxiety: insights from machine-learning and robust mediation-based models
Source: Front Psychiatry. 2026 May 18;17:1769001. doi: 10.3389/fpsyt.2026.1769001 (PMC13223371; doi:10.3389/fpsyt.2026.1769001)
Supplement: Supplementary file 1 [file DataSheet1.pdf]

Supplementary Material

1 Supplementary Figures

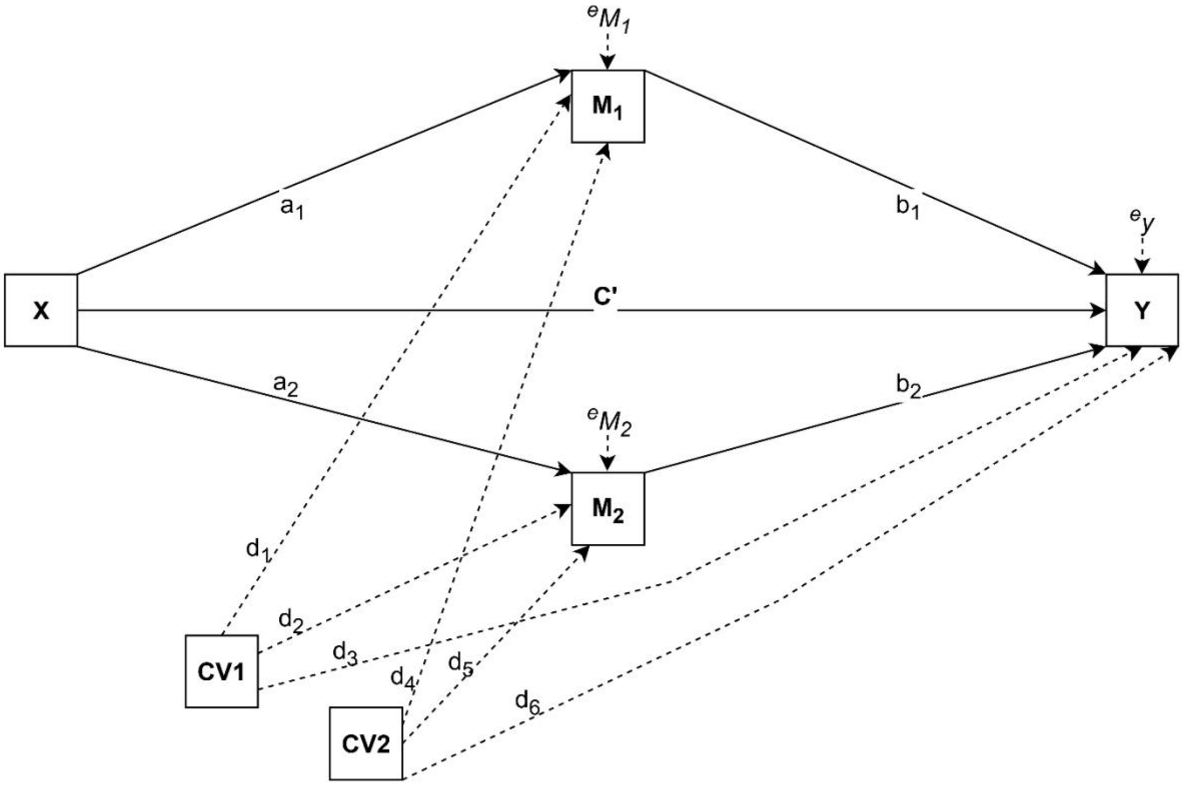

Figure S1. Theoretical Mediation Model. Mediation model for the relationship between psychological inflexibility (X) and an outcome measure (symptoms of generalized anxiety disorder; symptoms of anxiety; symptoms of stress) (Y), via two Mediators (dimensions of resilience) (M), adjusting for two relevant covariates (CV). a1, a2 represents the effects of X on M; b1, b2 represents the effects of M on Y; d1…d6 represents the effects of covariate(s) on M and on Y; e represents the error; c' is the direct effect of X on Y.

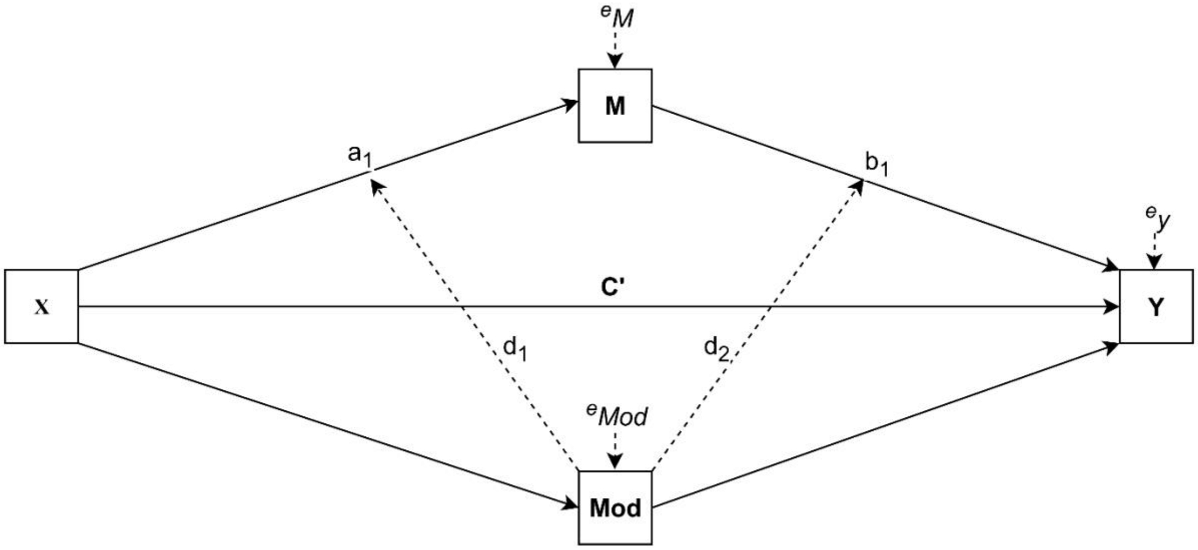

Figure S2. Theoretical Moderated Mediation Model. Moderated mediation model for the relationship between psychological inflexibility (X) and an outcome measure (symptoms of generalized anxiety disorder; symptoms of anxiety; symptoms of stress) (Y), via one Mediator (Perception of self dimension of resilience) (M), with this mediation association being moderated by a covariate (sex or history of psychiatric diagnosis) (Mod). a_1 represents the effects of X on M; b_1 represents the effects of M on Y; d_1 represents the effect of Mod on the association between X and M; d_2 represents the effect of Mod on the association between M and Y; e represents the error; c' is the direct effect of X on Y.

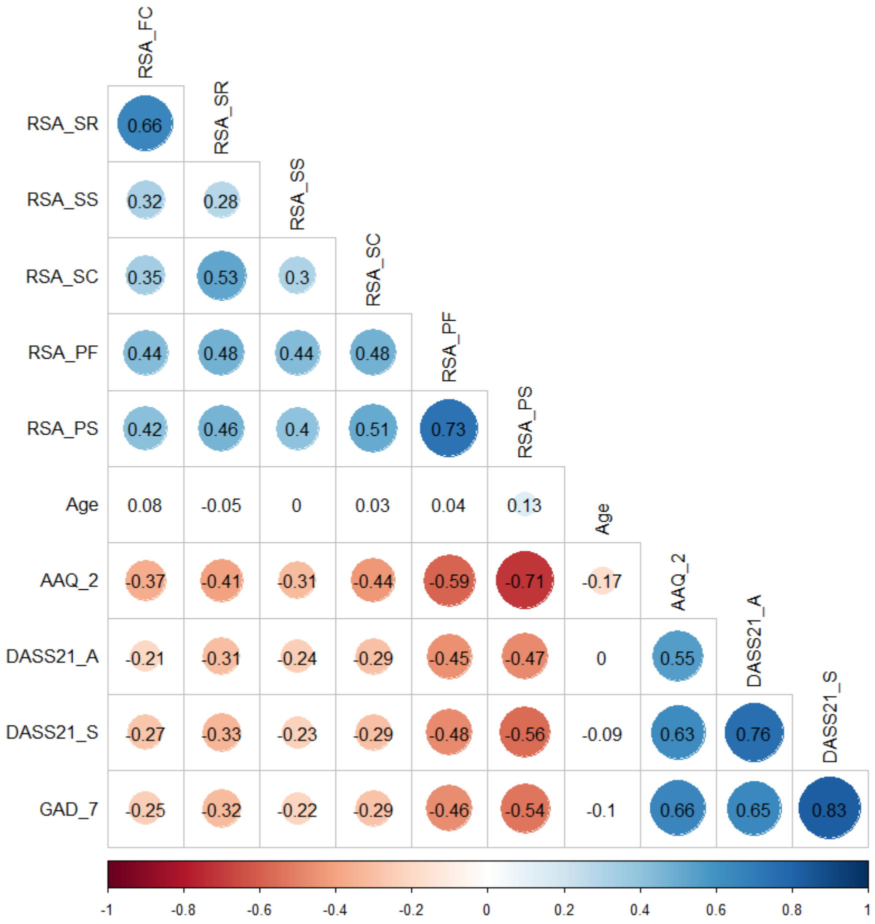

Figure S3. Pearson correlation matrix for all study variables. AAQ_2, Acceptance and Action Questionnaire-2; GAD_7, Generalized Anxiety Disorder Scale; DASS21, Depression, Anxiety, and Stress Scales; DASS21_A, Anxiety Scale; DASS21_S, Stress Scale; RSA, Resilience Scale for Adults; RSA_PS, Perception of self; RSA_PF, Planned future; RSA_SC, Social competence; RSA_SS, Structured style; RSA_FC, Family cohesion; RSA_SR, Social resources.

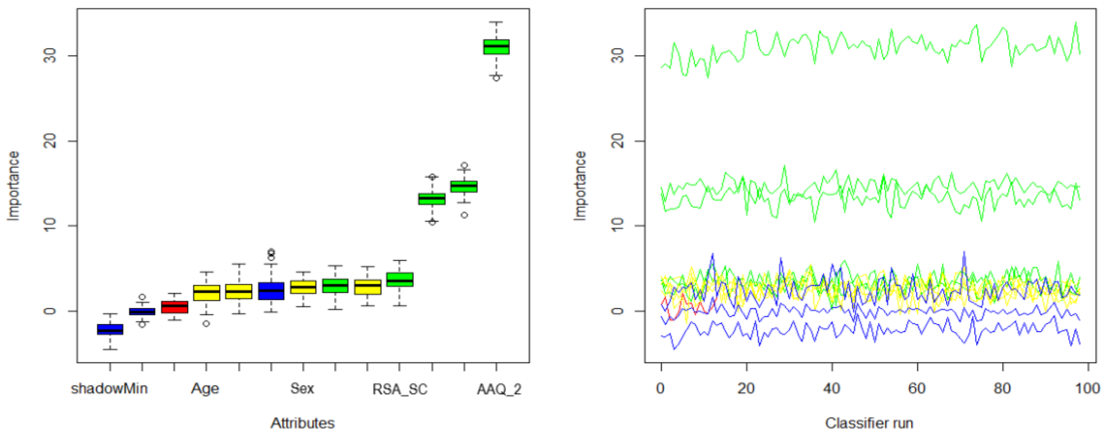

Figure S4. Boruta Results for the GAD-7 Outcome. HPD: History of Psychiatric Diagnosis; AAQ_2: Acceptance and Action Questionnaire-2; RSA: Resilience Scale for Adults. Variables confirmed as important in green [AAQ_2; RSA_PF (Planned future); RSA_PS (Perception of self); RSA_SC (Social competence); RSA_SS (Structured style)]; variables with tentative importance in yellow [Age; Sex; HPD; RSA_SR (Social resources)]; variables confirmed as unimportant in red [RSA_FC (Family cohesion)]; in blue are presented shadow variables (variables created by the algorithm to simulate original variables).

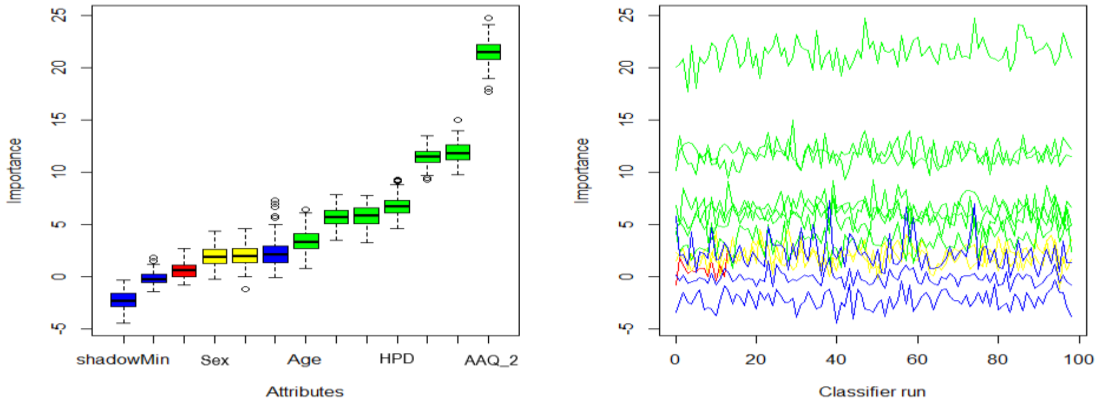

Figure S5. Boruta Results for the DASS21_Anxiety Outcome. HPD: History of Psychiatric Diagnosis; AAQ_2: Acceptance and Action Questionnaire-2; RSA: Resilience Scale for Adults. Variables confirmed as important in green [Age; HPD; AAQ_2; RSA_PF (Planned future); RSA_PS (Perception of self); RSA_SR (Social resources); RSA_FC (Family cohesion)]; variables with tentative importance in yellow [Sex; RSA_SC (Social competence)]; variables confirmed as unimportant in red [RSA_SS (Structured style)]; in blue are presented shadow variables (variables created by the algorithm to simulate original variables).

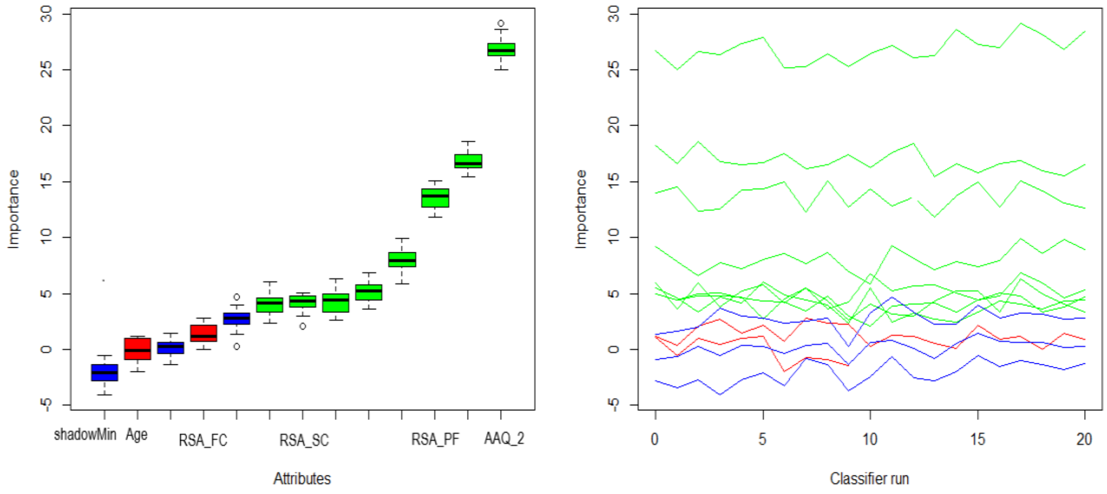

Figure S6. Boruta Results for the DASS21_Stress Outcome. HPD: History of Psychiatric Diagnosis; AAQ_2: Acceptance and Action Questionnaire-2; RSA: Resilience Scale for Adults. Variables confirmed as important in green [Sex; HPD; AAQ_2; RSA_PF (Planned future); RSA_PS (Perception of self); RSA_SR (Social resources); RSA_SS (Structured style); RSA_SC (Social competence)]; variables confirmed as unimportant in red [Age; RSA_FC (Family cohesion)]; in blue are presented shadow variables (variables created by the algorithm to simulate original variables).

frontiers

2 Supplementary Tables

Table S1. Pearson correlation for all study variables, r (*p*)

	GAD_7	DASS21_A	DASS21_S	Age	AAQ_2	RSA_PS	RSA_PF	RSA_SC	RSA_SS	RSA_FC	RSA_SR
GAD_7		0.648 (<.001)	0.825 (<.001)	-0.096 (.089)	0.657 (<.001)	-0.535 (<.001)	-0.465 (<.001)	-0.287 (<.001)	-0.219 (<.001)	-0.247 (<.001)	-0.318 (<.001)
DASS21_A	0.648 (<.001)		0.757 (<.001)	0.004 (.937)	0.554 (<.001)	-0.471 (<.001)	-0.453 (<.001)	-0.294 (<.001)	-0.242 (<.001)	-0.207 (<.001)	-0.305 (<.001)
DASS21_S	0.825 (<.001)	0.757 (<.001)		-0.086 (.128)	0.629 (<.001)	-0.562 (<.001)	-0.477 (<.001)	-0.294 (<.001)	-0.229 (<.001)	-0.271 (<.001)	-0.334 (<.001)
Age	-0.096 (.089)	0.004 (.937)	-0.086 (.128)		-0.171 (.002)	0.132 (.020)	0.043 (.446)	0.031 (.590)	-0.004 (.948)	0.084 (.140)	-0.045 (.424)
AAQ_2	0.657 (<.001)	0.554 (<.001)	0.629 (<.001)	-0.171 (.002)		-0.710 (<.001)	-0.587 (<.001)	-0.436 (<.001)	-0.314 (<.001)	-0.365 (<.001)	-0.409 (<.001)
RSA_PS	-0.535 (<.001)	-0.471 (<.001)	-0.562 (<.001)	0.132 (.020)	-0.710 (<.001)		0.732 (<.001)	0.505 (<.001)	0.403 (<.001)	0.418 (<.001)	0.464 (<.001)
RSA_PF	-0.465 (<.001)	-0.453 (<.001)	-0.477 (<.001)	0.043 (.446)	-0.587 (<.001)	0.732 (<.001)		0.477 (<.001)	0.440 (<.001)	0.437 (<.001)	0.476 (<.001)

	GAD_7	DASS21_A	DASS21_S	Age	AAQ_2	RSA_PS	RSA_PF	RSA_SC	RSA_SS	RSA_FC	RSA_SR
RSA_SC	-0.287 (<.001)	-0.294 (<.001)	-0.294 (<.001)	0.031 (.590)	-0.436 (<.001)	0.505 (<.001)	0.477 (<.001)		0.304 (<.001)	0.347 (<.001)	0.529 (<.001)
RSA_SS	-0.219 (<.001)	-0.242 (<.001)	-0.229 (<.001)	-0.004 (.948)	-0.314 (<.001)	0.403 (<.001)	0.440 (<.001)	0.304 (<.001)		0.324 (<.001)	0.281 (<.001)
RSA_FC	-0.247 (<.001)	-0.207 (<.001)	-0.271 (<.001)	0.084 (.140)	-0.365 (<.001)	0.418 (<.001)	0.437 (<.001)	0.347 (<.001)	0.324 (<.001)		0.656 (<.001)
RSA_SR	-0.318 (<.001)	-0.305 (<.001)	-0.334 (<.001)	-0.045 (.424)	-0.409 (<.001)	0.464 (<.001)	0.476 (<.001)	0.529 (<.001)	0.281 (<.001)	0.656 (<.001)	

Note. GAD_7, Generalized Anxiety Disorder Scale; DASS21, Depression, Anxiety, and Stress Scales; DASS21_A, Anxiety Scale; DASS21_S, Stress Scale; AAQ_2, Acceptance and Action Questionnaire-2; RSA, Resilience Scale for Adults; RSA_PS, Perception of self; RSA_PF, Planned future; RSA_SC, Social competence; RSA_SS, Structured style; RSA_FC, Family cohesion; RSA_SR, Social resources.

Table S2. Results from Boruta Algorithm About Important Variables for GAD-7 Outcome

Variable	meanlmp	medianlmp	minlmp	maxlmp	normHits	Decision
Age	2.25	2.31	-1.46	4.63	0.53	Tentative
Sex	2.83	2.88	0.57	4.65	0.61	Tentative
History of psychiatric diagnosis	2.30	2.36	-0.33	5.54	0.49	Tentative
Psychological inflexibility (AAQ-2)	30.98	31.11	27.39	33.97	1.00	Confirmed
Resilience – Perception of self (RSA)	14.66	14.69	11.33	17.18	1.00	Confirmed
Resilience – Planned future (RSA)	13.22	13.32	10.43	15.79	1.00	Confirmed
Resilience – Social competence (RSA)	3.66	3.57	0.64	5.93	0.81	Confirmed
Resilience – Structured style (RSA)	3.02	3.00	0.25	5.31	0.68	Confirmed
Resilience – Family cohesion (RSA)	0.51	0.65	-1.08	2.11	0.01	Rejected
Resilience – Social resources (RSA)	2.90	3.03	0.64	5.21	0.63	Tentative

Table S3. Results from Boruta Algorithm About Important Variables for DASS-21-Anxiety Outcome

Variable	meanImp	medianImp	minImp	maxImp	normHits	Decision
Age	3.43	3.36	0.82	6.43	0.82	Confirmed
Sex	1.96	1.94	-0.21	4.34	0.48	Tentative
History of psychiatric diagnosis	6.75	6.72	4.61	9.27	0.99	Confirmed
Psychological inflexibility (AAQ-2)	21.46	21.49	17.68	24.78	1.00	Confirmed
Resilience – Perception of self (RSA)	11.51	11.50	9.29	13.47	1.00	Confirmed
Resilience – Planned future (RSA)	11.95	11.86	9.77	14.98	1.00	Confirmed
Resilience – Social competence (RSA)	2.03	2.02	-1.16	4.57	0.42	Tentative
Resilience – Structured style (RSA)	0.71	0.65	-0.80	2.68	0.01	Rejected
Resilience – Family cohesion (RSA)	5.85	5.85	3.24	7.79	0.94	Confirmed
Resilience – Social resources (RSA)	5.77	5.71	3.47	7.84	0.94	Confirmed

Table S4. Results from Boruta Algorithm About Important Variables for DASS-21-Stress Outcome

Variable	meanImp	medianImp	minImp	maxImp	normHits	Decision
Age	-0.34	-0.06	-2.73	1.25	0.00	Rejected
Sex	3.72	3.66	1.35	5.74	0.80	Confirmed
History of psychiatric diagnosis	8.73	8.78	6.50	11.26	1.00	Confirmed
Psychological inflexibility (AAQ-2)	28.11	28.21	24.65	30.86	1.00	Confirmed
Resilience – Perception of self (RSA)	16.74	16.89	14.95	18.71	1.00	Confirmed
Resilience – Planned future (RSA)	13.31	13.10	10.70	16.04	1.00	Confirmed
Resilience – Social competence (RSA)	3.66	3.60	0.93	6.85	0.71	Confirmed
Resilience – Structured style (RSA)	4.51	4.58	1.18	6.82	0.86	Confirmed
Resilience – Family cohesion (RSA)	1.48	1.46	-0.49	3.86	0.02	Rejected
Resilience – Social resources (RSA)	4.17	4.13	1.01	6.72	0.86	Confirmed

Table S5. Variables Selection Based on Boruta Algorithm

OUTCOMES	FEATURE SELECTION WITH BORUTA ALGORITHM*<br>*Non-rejected predictors	Predictors included in final ML models
**GAD-7**	AGE; SEX; HPD; AAQ_2; RSA_PS; RSA_SC; RSA_PF; RSA_SS; RSA_SR	AGE; SEX; HPD; AAQ_2; RSA_PS; RSA_SC; RSA_PF; RSA_SS; RSA_SR
**DASS21_Anxiety**	AGE; SEX; HPD; AAQ_2; RSA_PS; RSA_SC; RSA_PF; RSA_SR; RSA_FC	AGE; SEX; HPD; AAQ_2; RSA_PS; RSA_SC; RSA_PF; RSA_SR; RSA_FC
**DASS21_Stress**	SEX; HPD; AAQ_2; RSA_PS; RSA_SC; RSA_PF; RSA_SS; RSA_SR	SEX; HPD; AAQ_2; RSA_PS; RSA_SC; RSA_PF; RSA_SS; RSA_SR

Note. HPD, History of Psychiatric Diagnosis; AAQ_2, Acceptance and Action Questionnaire-2; RSA, Resilience Scale for Adults; RSA_PS, Perception of self; RSA_PF, Planned future; RSA_SC, Social competence; RSA_SS, Structured style; RSA_FC, Family cohesion; RSA_SR, Social resources.

Table S6. Full statistical results for parallel mediation model with Generalized Anxiety Disorder symptoms (GAD_7)

	Generalized Anxiety Disorder symptoms (GAD_7)						
**Paths and effects**	β (data)	β (boot)	SE	*Z*-value	*p*-value	Robust R^2	95%CI
Outcome: RSA_PS						0.545	
AAQ_2 → RSA_PS	-0.518	-0.518	0.033	-15.466	< 0.001		
Age → RSA_PS	0.011	0.011	0.029	0.368	0.713		
Outcome: RSA_PF						0.360	
AAQ_2 → RSA_PF	-0.342	-0.342	0.030	-11.537	< 0.001		
Age → RSA_PF	-0.037	-0.037	0.025	-1.492	0.136		
Outcome: GAD-7						0.544	
RSA_PS → GAD_7	-0.080	-0.078	0.053	-1.468	0.142		
RSA_PF → GAD_7	-0.112	-0.113	0.059	-1.896	0.058		
Age → GAD-7	-0.003	-0.003	0.018	-0.151	0.880		
Total effect of AAQ_2 on GAD_7	0.355	0.354	0.027	12.950	< 0.001		
Direct effect of AAQ_2 on GAD_7	0.275	0.275	0.039	6.975	< 0.001		
**Indirect effects of AAQ_2 on GAD_7**							
Total (RSA_PS + RSA_PF)	0.079	0.079			0.001		[0.034, 0.128]
RSA_PS	0.041	0.040			0.117		[-0.011, 0.099]
RSA_PF	0.038	0.039			0.067		[-0.003, 0.080]

Note. GAD_7, Generalized Anxiety Disorder Scale; SE, Standard error; CI, Confidence interval; AAQ_2, Acceptance and Action Questionnaire-2; RSA, Resilience Scale for Adults; RSA_PS, Perception of self; RSA_PF, Planned future; GAD_7, Generalized Anxiety Disorder Scale; → Direct effect on. Robust mediation models have been run with 5000 bootstrap replicates.

Table S7. Full statistical results for parallel mediation model with anxiety symptoms (DASS21_A)

	Anxiety symptoms (DASS21_A)						
**Paths and effects**	β (data)	β (boot)	SE	*Z*-value	*p*-value	Robust R^2	95%CI
Outcome: RSA_PS						0.545	
AAQ_2 → RSA_PS	-0.523	-0.523	0.032	-16.117	< 0.001		
HPD → RSA_PS	0.314	0.303	0.828	0.366	0.714		
Outcome: RSA_PF						0.372	
AAQ_2 → RSA_PF	-0.351	-0.351	0.030	-11.880	< 0.001		
HPD → RSA_PF	1.485	1.458	0.824	1.770	0.077		
Outcome: DASS21_A						0.266	
RSA_PS → DASS21_A	0.038	0.038	0.029	1.315	0.189		
RSA_PF → DASS21_A	-0.080	-0.079	0.037	-2.119	0.034		
HPD → DASS21_A	0.769	0.773	0.318	2.431	0.015		
Total effect of AAQ_2 on DASS21_A	0.075	0.075	0.018	4.166	< 0.001		
Direct effect of AAQ_2 on DASS21_A	0.066	0.067	0.021	3.180	0.001		
**Indirect effects of AAQ_2 on DASS21_A**							
Total (RSA_PS + RSA_PF)	0.008	0.008			0.414		[-0.012, 0.033]
RSA_PS	-0.020	-0.020			0.214		[-0.049, 0.010]
RSA_PF	0.028	0.028			0.027		[0.004, 0.056]

Note. DASS21, Depression, Anxiety, and Stress Scales; DASS21_A, Anxiety Scale; SE, Standard error; CI, Confidence interval; AAQ_2, Acceptance and Action Questionnaire-2; HPD, History of Psychiatric Diagnosis; RSA, Resilience Scale for Adults; RSA_PS, Perception of self; RSA_PF, Planned future; GAD_7, Generalized Anxiety Disorder Scale; → Direct effect on. Robust mediation models have been run with 5000 bootstrap replicates

Table S8. Full statistical results for parallel mediation model with stress symptoms (DASS21_S)

	Stress symptoms (DASS21_S)						
**Paths and effects**	β (data)	β (boot)	SE	*Z*-value	*p*-value	Robust R^2	95%CI
Outcome: RSA_PS						0.544	
AAQ_2 → RSA_PS	-0.520	-0.520	0.032	-16.044	< 0.001		
Sex → RSA_PS	0.105	0.108	0.667	0.162	0.872		
Outcome: RSA_PF						0.356	
AAQ_2 → RSA_PF	-0.337	-0.337	0.030	-11.246	< 0.001		
Sex → RSA_PF	0.045	0.052	0.622	0.083	0.934		
Outcome: DASS21_S						0.484	
RSA_PS → DASS21_S	-0.155	-0.152	0.055	-2.765	0.006		
RSA_PF → DASS21_S	-0.080	-0.082	0.056	-1.458	0.145		
Sex → DASS21_S	1.066	1.060	0.429	2.471	0.013		
Total effect of AAQ_2 on DASS21_S	0.303	0.302	0.030	10.060	< 0.001		
Direct effect of AAQ_2 on DASS21_S	0.195	0.195	0.040	4.869	< 0.001		
**Indirect effects of AAQ_2 on DASS21_S**							
Total (RSA_PS + RSA_PF)	0.108	0.107			0.0001		[0.058, 0.160]
RSA_PS	0.081	0.079			0.004		[0.027, 0.141]
RSA_PF	0.027	0.028			0.143		[-0.009, 0.067]

Note. DASS21, Depression, Anxiety, and Stress Scales; DASS21_S, Stress Scale; SE, Standard error; CI, Confidence interval; AAQ_2, Acceptance and Action Questionnaire-2; HPD, History of Psychiatric Diagnosis; RSA, Resilience Scale for Adults; RSA_PS, Perception of self; RSA_PF, Planned future; GAD_7, Generalized Anxiety Disorder Scale; → Direct effect on. Robust mediation models have been run with 5000 bootstrap replicates

Table S9. Moderated Mediation Models Analysis for Sex and History of Psychiatric Diagnosis Groups

	Generalized Anxiety Disorder symptoms (GAD_7)			Anxiety symptoms (DASS21_A)			Stress symptoms (DASS21_S)		
Groups and Model Parameters	β	95% CI	*p*	β	95% CI	*p*	β	95% CI	*p*
Males									
ACME	0.05	-0.00 – 0.12	0.08	0.04	0.00 – 0.08	0.04	0.07	0.02 – 0.15	0.004
ADE	0.29	0.14 – 0.37	< .001	0.04	-0.03 – 0.11	0.27	0.19	0.07 – 0.27	< .001
Total effect	0.33	0.20 – 0.40	< .001	0.07	0.01 – 0.13	0.01	0.26	0.14 – 0.32	< .001
Prop. Mediated	0.14	-0.00 – 0.40	0.08	0.50	0.13 – 4.37	0.05	0.27	0.09 – 0.66	0.004
Females									
ACME	0.05	-0.01 – 0.10	0.08	0.04	0.00 – 0.08	0.04	0.07	0.03 – 0.13	0.004
ADE	0.30	0.22 – 0.38	< .001	0.17	0.12 – 0.22	< .001	0.23	0.16 – 0.30	< .001
Total effect	0.35	0.30 – 0.40	< .001	0.21	0.16 – 0.26	< .001	0.30	0.25 – 0.35	< .001
Prop. Mediated	0.14	-0.01 – 0.31	0.08	0.18	0.01 – 0.37	0.04	0.24	0.09 – 0.41	0.004
Test of ACME(covariates.1) – ACME(covariates.2) = 0			0.90			0.90			0.96
Without history of psychiatric diagnosis									
ACME	0.09	-0.02 – 0.18	0.12	0.06	-0.00 – 0.13	0.08	0.12	0.04 – 0.20	< .001

	Generalized Anxiety Disorder symptoms (GAD_7)			Anxiety symptoms (DASS21_A)			Stress symptoms (DASS21_S)		
Groups and Model Parameters	β	95% CI	*p*	β	95% CI	*p*	β	95% CI	*p*
ADE	0.27	0.13 – 0.44	< .001	0.10	-0.01 – 0.18	0.05	0.23	0.12 – 0.31	< .001
Total effect	0.36	0.24 – 0.45	< .001	0.16	0.09 – 0.22	< .001	0.35	0.25 – 0.43	< .001
Prop. Mediated	0.24	-0.04 – 0.60	0.12	0.36	0.00 – 1.10	0.08	0.35	0.15 – 0.60	< .001
With history of psychiatric diagnosis									
ACME	0.07	-0.01 – 0.16	0.12	0.05	-0.00 – 0.11	0.08	0.10	0.04 – 0.18	< .001
ADE	0.26	0.14 – 0.42	< .001	0.19	0.10 – 0.29	< .001	0.16	0.06 – 0.29	< .001
Total effect	0.34	0.23 – 0.44	< .001	0.24	0.16 – 0.33	< .001	0.27	0.16 – 0.37	< .001
Prop. Mediated	0.22	-0.03 – 0.54	0.12	0.21	-0.01 – 0.48	0.08	0.39	0.16 – 0.73	< .001
Test of ACME(covariates.1) – ACME(covariates.2) = 0			0.82			0.82			0.90

Note. ACME, Average Causal Mediation Effect [total effect - direct effect]; ADE, Average Direct Effect [total effect - indirect effect]; Total effect: Direct (ADE) + Indirect (ACME); Prop. Mediated: Conceptually ACME / Total effect. The variables *sex* and *history of psychiatric diagnosis* were subject to Propensity Score Matching (more details in the statistical analysis section).
